# Liver Disease in Common Variable Immunodeficiency: Current Evidence and Knowledge Gaps

**DOI:** 10.3390/ijms27031518

**Published:** 2026-02-03

**Authors:** Irena Nedelea, Oana Nicoara-Farcau, Bogdan Procopet, Horia Stefanescu, Corina Radu, Radu Balan, Ana-Maria Fit, Ioana Rusu, Diana Deleanu

**Affiliations:** 1 Allergy and Immunology Discipline, Iuliu Hațieganu University of Medicine and Pharmacy, 400012 Cluj-Napoca, Romania; irena.nedelea@umfcluj.ro (I.N.);; 2 Allergy and Clinical Immunology Outpatient Clinic, Octavian Fodor Regional Institute of Gastroenterology and Hepatology, 400158 Cluj-Napoca, Romania; 3 Gastroenterology and Hepatology Discipline, Iuliu Hațieganu University of Medicine and Pharmacy, 400012 Cluj-Napoca, Romania; 4 Hepatology Department, Octavian Fodor Regional Institute of Gastroenterology and Hepatology, 400158 Cluj-Napoca, Romania; 5 Pathology Department, Octavian Fodor Regional Institute of Gastroenterology and Hepatology, 400158 Cluj-Napoca, Romania

**Keywords:** common variable immunodeficiency, liver disease, porto-sinusoidal vascular disorder

## Abstract

Common variable immunodeficiency (CVID) is the most prevalent symptomatic primary immunodeficiency or inborn error of immunity (IEI) encountered in clinical practice. Characterized by a remarkably broad clinical spectrum, CVID presents with phenotypes spanning from “infection only” to significant non-infectious complications. The frequent overlap between these classifications underscores that their distinction is more accurately viewed as a continuous spectrum, rather than a binary categorization. CVID-associated liver disease is a significant source of morbidity, yet often poses diagnostic challenges due to its insidious and clinically silent nature, typically becoming apparent only upon the development of complications. Manifestations range from abnormal liver tests to irreversible organ damage, with reports including granulomas, autoimmune hepatitis, fibrosis, and porto-sinusoidal vascular disorder (PSVD). Regenerative nodular hyperplasia (RNH), commonly associated with PSVD, is a frequent histopathological finding. Management requires a multidisciplinary approach, including cause-directed immunosuppression and supportive treatment for non-cirrhotic portal hypertension. Despite significant advances in comprehending CVID-associated liver involvement, substantial gaps persist concerning its pathogenesis, its optimal management, and the correlation between histological findings and clinical outcomes. A heightened awareness of CVID-associated liver disease is paramount for multidisciplinary teams across IEI centers. Furthermore, given its prevalence, its insidious clinical phenotype until advanced complications, and the significant diagnostic delay and underdiagnosis, such awareness is critical across a broader range of medical specialties. In this paper, we aim to consolidate current knowledge regarding CVID-related liver disease, examining its clinical presentation, recent genetic and pathogenetic advancements along with current diagnostic methodologies, and therapeutic strategies.

## 1. Overview of Common Variable Immunodeficiency

Common variable immunodeficiency (CVID) is the most frequent symptomatic primary antibody deficiency in the adult population, with an estimated incidence ranging between 1 in 10,000 and 1 in 50,000 individuals and reported prevalence estimates ranging from 0.001 to 6.9 per 100,000 individuals [1,2,3,4]. The diagnostic delay of CVID remains a significant global challenge, with patients often experiencing several years of delayed diagnosis across different regions, adversely impacting disease management and outcomes [5,6,7,8]. Reports indicate that each year of delay in diagnosis is associated with a 1.7% increase in mortality risk [9]. The majority of CVID cases are diagnosed in adulthood, with published series commonly finding approximately 60–80% of patients presenting after 18 years of age. The median age at diagnosis is frequently in the 20s–30s, although a wide age range and occasional later-life diagnoses are observed [1,7,9].

Although considerable challenges exist in reaching a consensus on the diagnosis of such a complex disease, and identifying a single strategy that adequately encompasses the heterogeneity of CVID remains difficult, most clinicians adhere to the diagnostic hallmarks of the European Society for Immunodeficiencies (ESID) Registry (as outlined at https://esid.org/...rking-Party/Diagnosis-criteria, accessed on 30 January 2026), and of the International Consensus Document (ICON) [10,11].

Along with advances in understanding the genetic causes of inborn errors of immunity (IEI), CVID is now regarded as a heterogeneous group of conditions where a robust genetic basis is identified in some cases [2,12]. CVID includes monogenic forms of the disease, which account for approximately 20% of cases in non-consanguineous cohorts and about 70% in consanguineous cohorts. To date, over 60 genes have been characterized as monogenic CVID, with additional genes being associated with a CVID-like phenotype. Given that hypogammaglobulinemia is a defining characteristic of CVID, it is not surprising that many identified genes associated with the condition are involved in B lymphocyte survival, proliferation, development, maturation, and function. However, a subset of CVID patients, despite lacking an identified genetic defect, also exhibits a markedly reduced number of B lymphocytes. In the majority of instances, the etiology, whether oligogenic or resulting from polygenic interactions, remains insufficiently understood. In addition, epigenetic and somatic factors contributing to CVID susceptibility have gained increasing recognition and momentum in recent research [13,14]. CVID often manifests in adulthood despite its genetic contributions, due to variable penetrance, multifactorial etiology, and cumulative or co-triggered immune dysfunction [13].

The complexity of CVID pathogenesis remains a topic of intense investigation. CVID is a broad diagnostic label encompassing symptomatic primary antibody deficiencies, coupled with immune-regulatory abnormalities that drive systemic immune activation, T-cell dysfunction, and the dysregulation of several innate immune cells. These abnormal immune responses culminate in a persistent, systemic proinflammatory milieu. The contemporary view frames CVID as a heterogeneous group of immune-dysregulatory disorders, rather than merely as an infectious consequence of hypogammaglobulinemia [15]. In addition, the CVID population has a defective intestinal microbiome signature, which is suspected to be an important factor in the activation of T lymphocytes and the monocyte–macrophage system, as it permits the passage of pathogens and microbial toxins from the gut into the systemic circulation [16].

Reports indicate that roughly 70% of patients exhibit one or more of the following: autoimmune disease, enteropathy, polyclonal lymphocytic infiltration (including splenomegaly and granulomas), or malignancy [9,10,11]. A large-cohort study of CVID spanning four decades, which reported an overall mortality rate of 19.6%, demonstrated that patients with non-infectious complications face an eleven-fold higher risk of mortality, with lung disease being the leading cause of death, followed by lymphoma and liver disease [5]. Owing to the overlap among these phenotypic classifications, it is imperative to view their distinction as a continuous clinical spectrum, rather than a binary division.

Lifelong IgRT is indispensable for managing CVID, as it mitigates the risk and severity of infectious complications, alongside an individualised approach to infection prophylaxis, organ-directed therapies, and surveillance for complications. Hematopoietic stem cell transplantation (HSCT) may be considered on a case-by-case basis but is associated with a substantially increased risk of infectious complications [17]. While IgRT is the mainstay treatment for CVID with infectious complications, evidence supporting its impact on non-infectious complications is currently limited [1,5,7].

Liver involvement is a notable source of morbidity in CVID patients and poses diagnostic challenges due to its often clinically silent nature. This review aims to synthesize current knowledge regarding CVID-related liver disease, examining its clinical presentation, recent genetic and pathogenetic advancements along with current diagnostic methodologies, and therapeutic strategies, and concurrently identify remaining knowledge gaps.

## 2. Liver Disease in CVID

### 2.1. Clinical Spectrum and Histopathological Features

Liver disease in CVID is often silent, becoming clinically evident only with the development of complications, which leads to delayed diagnosis in many cases and carries a high risk of mortality and morbidity [18,19,20].

The reported prevalence of liver disease in CVID varies depending on the diagnostic criteria considered (cohort, selection, disease definition, laboratory, clinical, imaging, or histopathological parameters), ranging from 5% to 79% of cases [21,22]. A study that included 141 CVID patients documented the presence of liver disease in 46 patients (33%). The liver disease in this study was defined as “imaging signs of chronic liver parenchymal disease, excluding steatosis” [23]. Another study involving 77 patients observed liver disease in 33.8% of cases. Liver involvement was defined by hepatic fibrosis assessed through elastography [24]. A study that used histopathological criteria to define liver disease reported a prevalence of 9.3% [25].

Liver involvement varies widely in clinical presentation, from abnormal test results to irreversible organ damage and extrahepatic complications. Reports in CVID patients include intraparenchymal granulomas, autoimmune hepatopathy, fibrosis and/or perisinusoidal lymphocytosis, primary biliary cholangitis, liver cirrhosis, primary sclerosing cholangitis, regenerative nodular hyperplasia (RNH) and rupture of RNH masses, porto-sinusoidal vascular disorder (PSVD), and its complications such as esophageal and gastric varices, gastrointestinal hemorrhage due to variceal rupture, ascites, spontaneous bacterial peritonitis, hepatopulmonary syndrome, portopulmonary hypertension, hepatic encephalopathy, as well as hypersplenism and secondary neutropenia, with a high risk of fulminant infections [20,25,26,27]. Most often, liver disease in CVID is accompanied by other systemic manifestations. PSVD is mainly present in CVID patients with non-infectious complications such as granulomatous and interstitial lymphocytic pulmonary disease (GLILD), persistent benign lymphadenopathy, splenomegaly, enteropathy, granulomatous disease, and cytopenias [21]. The main liver manifestations of CVID are summarized in Table 1.

Differential diagnosis includes liver involvement from other causes (medication-induced, alcohol-related liver damage, metabolic dysfunction-associated liver disease, etc.), infections, parasitic infestations, primary or secondary cancers, benign liver lesions, and vascular diseases progressing with hepatic or portal vein thrombosis. Although inherited or acquired thrombophilia is considered an important cause of PSVD, studies have shown that its prevalence in PSVD patients is similar to that of the general population. Additionally, RNH predisposes one to thrombosis and microvascular disease. RNH, in the absence of CVID, is mainly found in association with drug exposure such as Diadanosine, Oxaliplatine or Azathioprine. PSVD represents a common final pathway for a diverse spectrum of underlying conditions, encompassing medication-induced etiologies, genetic (e.g., familial/congenital vascular disorders) and hematologic predispositions, immune-mediated diseases (including other IEIs or autoimmune conditions), and other rare etiologies (such as human immunodeficiency virus infection or recurrent gastrointestinal infections) [22,27,28,29].

**Table 1:** Main liver presentations in CVID.

Liver Manifestation	Histological and Clinical Features
PSVD (dominant liver pathology) [20,21,22,23,24,25,26,27,28,30,31,32]	Specific histological signs: - Obliterative portal venopathy - Nodular regenerative hyperplasia - Incomplete septal fibrosis Non-specific histological lesions: - Portal tract abnormalities (multiplication, dilatation of arteries, periportal vascular channels, abberant vessels) - Non-zonal sinusoidal dilation - Architectural disruptions (irregular distribution of the portal tracts and central veins) - Mild perisinusoidal fibrosis - Microvascular thrombosis Specific signs of portal hypertension (PH): - Eso-gastric or ectopic varices (and variceal efraction) - Porto-systemic collaterals at imaging - Non-specific signs of PH - Ascites - Platelet count < 15.000 cells/microL - Splenomegaly > 130 cm in the largest axis Associated clinical features/complications: - Rupture of RNH masses - Hepatomegaly/splenomegaly - Digestive hemorrhage from variceal effraction - Spontaneous bacterial peritonitis - Hepatic encephalopathy - Hepatopulmonary syndrome (arterial deoxygenation and intrapulmonary vascular dilatation, pulmonary arterio-venous shunting, orthodeoxia and platypnoea) - Hepatorenal syndrome - Hypersplenism, trombocytopenia and secondary neutropenia, with a high risk of fulminant infections - Other CVID-associated conditions (enteropathy, GLILD), autoimmune cytopenia, benign lymphoproliferative disease)
Granulomatous liver disease (granulomatous disease in CVID ranges from 8–22%, with liver involvement occuring in a subset of these patients) [33,34,35]	Histological features: - Formation of non-caseating granulomas, often sarcoid like, but displaying distinct features such as smaller, less circumscribed lesions, fewer multinucleated giant cells, and minimal fibrosis - Granulomatous lesions are composed of highly activated immune B and T cells, macrophages, and neutrophils, and attributable to underlying chronic immune dysregulation and lymphoid hyperplasia Clinical features: - Non-specific constitutional symptoms (fever, fatigue, malaise, anorexia, and weight loss) - Liver-specific signs (hepatomegaly, splenomegaly, jaundice, pruritus) - CVID-associated clinical features (severe recurrent infections, lymphadenopathy, enteropathy, autoimmune cytopenia, granulomatous disease of the spleen, lung or skin)
Autoimmune hepatitis (AIH) [6,36,37]	Histological features: - Active interface hepatitis associated with a lympho-plasmacytic infiltrate, progressing to periportal and potentially bridging fibrosis (plasma cells are typically absent, distinghuishing CVID-associated AIH from classic AIH) Clinical features are non-specific, constitutional symptoms, associated with typical signs of liver involvement; diagnosis requires liver biopsy (as per expert consensus), positive AIH immunoserology (it is noteworthy that discrepancy between pathohistological findings and immunoserology is common, with lesions detectable histologically even when serological markers are negative, due to the immunological defects consistent with CVID (i.e., impaired B-cell differentiation and hypogammaglobulinemia); thus, classic serological markers of AIH are often negative or unreliable in CVID), and exclusion of other causes of hepatitis, along with CVID associated clinical features
Cryptogenic cirrhosis [6,38]	Histological features: - Irreversible and diffuse bridging fibrosis - Formation of regenerative nodules - Complete distortion of the normal liver architecture - Clinical features: - Non-specific systemic symptoms - Signs of liver failure and PH - Associated CVID - The absence of other causes of liver disease

AIH: autoimmune hepatitis; CVID: common variable immunodeficiency; i.e.,: id est; GLILD: granulomatous and interstitial lymphocytic pulmonary disease; microL: microliter; PH: portal hypertension; PSVD: porto-sinusoidal vascular disorder; RNH: regenerative nodular hyperplasia.

**Figure 1:**
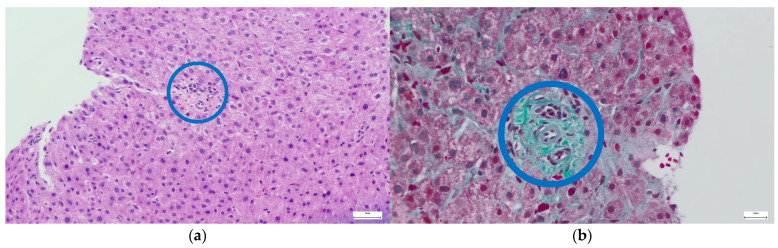
Portal tracts with venopenia (blue circle): (**a**) Hematoxylin & Eosin staining, (**b**) Masson’s trichrome staining.

**Figure 2:**
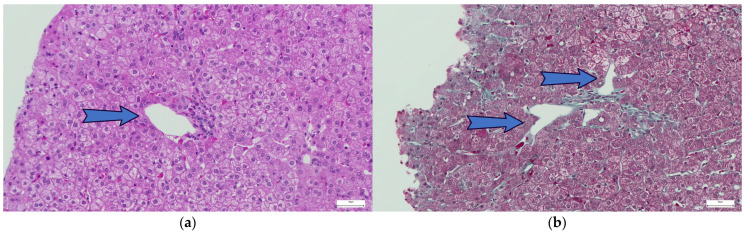
Dilated venules: (**a**) Hematoxylin & Eosin staining; (**b**) Masson’s trichrome staining. The arrows point to the two types of dilated venules.

**Figure 3:**
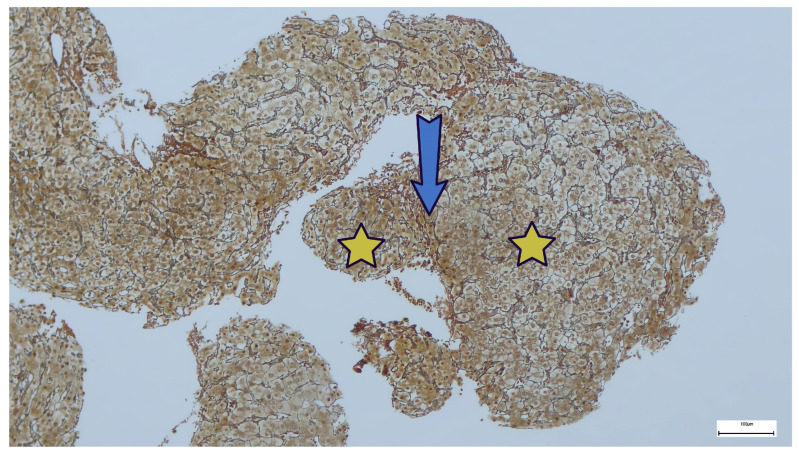
Nodular regenerative hyperplasia: Gomori staining shows hepatocytic nodules (without fibrosis) composed of thickened trabeculae (yellow stars) and separated by thinner hepatocyte trabeculae (blue arrow).

Among the hepatic complications associated with CVID, the most prevalent are granulomatous disease (≤36%) and RNH, which is associated with PSVD and found in 44–86% of cases [27,28]. The definitive diagnosis of RNH is based on histopathological criteria, using hematoxylin-eosin and reticulin staining. RNH is defined as “the presence of multiple hepatocellular nodules of 1–2 mm, with a central part consisting of hypertrophic hepatocytes, with reduced, incomplete, or absent fibrous septa, separated or surrounded at the periphery by atrophic hepatocytes that compress the sinusoids” (Figure 1, Figure 2 and Figure 3; images are sourced from the authors’ internal collection).

Obliterative portal venopathy (OPV) is another hallmark histological feature of PSVD. It is characterized by a progressive thickening of small intrahepatic portal venules, culminating in luminal stenosis or complete occlusion. Perivenular fibrosis frequently accompanies these changes, further promoting obliteration, while local microthrombosis exacerbates hemodynamic impairment. These pathological alterations ultimately result in PH, crucially, in the absence of hepatic cirrhosis. Thus, RNH, OPV, and fibrosis with incomplete septa are essential “specific” features of histopathological diagnosis. RNH and OPV often coexist. The most common “non-specific” sign is sinusoidal dilation, observed in nearly half of the cases. RNH is associated with PSVD and non-cirrhotic PH (NCPH) [27].

The basic histopathological diagnostic criteria of PSVD in CVID are [30]:

- Absence of cirrhosis: liver architecture is preserved without the broad fibrous septa and nodular transformation characteristic of cirrhosis.
- Presence of specific portal vascular lesions, which may include:
  - OPV: partial or complete loss of portal vein branches, often with replacement by small, thin-walled vessels or fibrous tissue.
  - NRH: diffuse transformation of hepatic parenchyma into small regenerative nodules without significant fibrosis. This is a hallmark lesion in CVID-related PSVD and is frequently observed in liver biopsies from affected patients.
  - Incomplete septal fibrosis/cirrhosis: thin, incomplete fibrous septa that do not form true cirrhotic nodules.
- Additional suggestive features (not required but supportive):
  - Herniated portal veins into the periportal parenchyma.
  - Hypervascularized portal tracts or abnormal periportal vessels.
  - Sinusoidal dilatation, especially in the absence of congestion.
  - Regenerative hepatocyte changes.

The histopathological evaluation of liver biospy focuses on identifying these characteristic features, along with sinusoidal lymphocytic infiltration (predominantely T cells), and sinusoidal endothelial changes (e.g., increased CD34 expression) [39]. Plasma cells are typically absent, and granulomatous inflammation may be present in some cases [33,34,35,40,41,42,43].

Diagnosing PSVD is challenging and requires a high index of clinical suspicion and interpretation by experienced pathologists [27]. Its diagnosis demands a multifaceted approach, integrating clinical, vascular, etiological, and histopathological parameters, complemented by specific radiological or hemodynamic features. Notably, PSVD is recognized as a distinct subset of NCPH, defined by specific diagnostic criteria. Current guidelines define PSVD as:

- liver biopsy sample (of at least 20 mm) without cirrhosis and one specific sign for PH or one histological lesion specific for PSVD; or
- liver biopsy sample (of at least 20 mm) without cirrhosis and both one sign not specific for PH and one histological lesion not specific for PSVD [27].

### 2.2. Pathogenetic Mechanisms of PSVD in CVID

**Figure 4:**
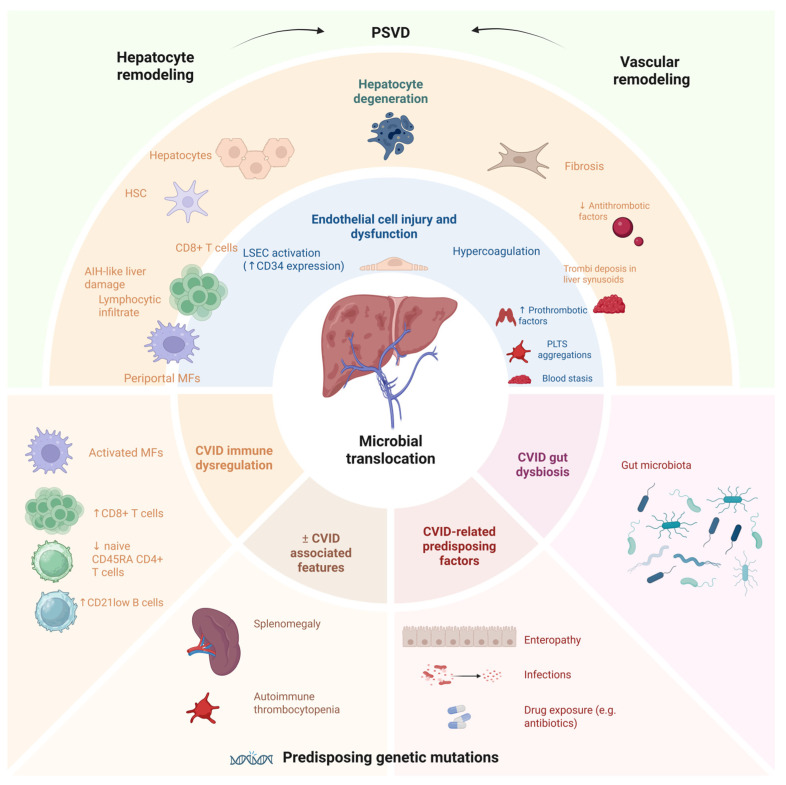
The multifactorial pathophysiology of PSVD seems to be primarily driven by CVID immune dysregulation and gut dysbiosis. Microbial translocation appears to be a key trigger for a hepatic immune attack on liver sinusoidal endothelial cells (LSECs) and hepatocytes. Activated hepatic stellate cells (HSCs) induce fibrogenesis and hepatocyte death. Liver fibrosis and microvascular inflammation within this cycle promote hypercoagulation (due to impaired antithrombotic and enhanced prothrombotic factors). The vascular and hepatocyte remodeling contribute to NRH, hemodynamic alterations, and a persistent prothrombotic state, with possible aggravating factors like splenomegaly and thrombocytopenia. A possible genetic landscape for PSVD susceptibility in CVID is under investigation.

The pathogenic mechanisms of RNH and PSVD are insufficiently clarified. Models of pathogenesis involve chronic immune-mediated sinusoidal and hepatocyte injury, endothelial activation, and microvascular remodeling, with genetic and immunophenotypic factors modulating susceptibility and disease progression [27,31,39,44,45,46]. Figure 4 outlines the possible pathophysiological events leading to liver injury, particularly to PSVD in CVID.

NRH and PSVD in CVID are driven by immune-mediated mechanisms, primarily involving lymphocytic infiltration and endothelial injury. In CVID, NRH is characterized by diffuse transformation of hepatic parenchyma into regenerative nodules without significant fibrosis. The pathogenetic mechanism is thought to involve chronic, low-grade lymphocytic infiltration—predominantly cytotoxic T cells—within hepatic sinusoids, leading to endothelial injury, microvascular occlusion, and subsequent parenchymal atrophy and nodular regeneration [39,45]. This process is supported by histological findings of sinusoidal T-cell infiltration, increased CD34 expression (reflecting sinusoidal endothelial activation), and pericellular elastosis, with progression to fibrosis in some cases [39,45]. In many cases of CVID and PSVD, there is a CD8+ T lymphocytic infiltrate at the hepatic level, particularly in the sinusoidal endothelial cells, suggesting a direct cytotoxic attack by these cells that leads to apoptosis and tissue damage [22,32,47,48,49]. An autoimmune attack of the liver is suspected, based on the observation that some CVID patients with RNH show histopathological features similar to AIH, characterized by portal inflammatory infiltrate, bridging necrosis, and the absence of plasma cells, and are clinically associated with a more severe pattern of liver involvement and biochemically with impaired hepatic excretory function [27]. Electron microscopy and immunohistochemistry demonstrate hepatocyte degeneration and aberrant von Willebrand factor expression in sinusoidal endothelial cells, further implicating endothelial dysfunction in NRH pathogenesis [50].

Endothelial dysfunction and microvascular inflammation are associated with platelet aggregates within the sinusoids and with an increased number of Toll-like Receptor 4 (TLR4)-positive macrophages, which are crucial pattern recognition receptor that detects bacterial components and danger signals, triggering strong inflammatory responses. Microvascular changes and endothelial dysfunction are linked to increased gut-derived endotoxins (notably lipopolysaccharide, LPS), which drive local inflammation via the LPS–TLR4 pathway, promoting platelet aggregation and microthrombi formation [51,52]. Abnormalities of the gut–liver axis, supported by the observation that persistent enteropathy and intestinal dysbiosis are common in the CVID population are another contributing factor. It is thought that recurrent digestive infections are a contributing factor to the pathogenesis of PSVD in CVID patients. These conditions predispose to microbial translocation, facilitating the passage of pathogenic agents and endotoxins to extraintestinal sites, including the hepatic portal circulation. The evaluation of endotoxemia through the measurement of plasma LPS may be useful to document bacterial translocation [9,16,53,54,55,56,57].

Recent studies show that the liver, as the primary gateway between the gut and systemic circulation, hosts periportal macrophages that defend against commensal-driven hepatic inflammation. A periportal subset of immunosuppressive macrophages, characterized by high interleukin-10 (IL-10) and MARCO (Macrophage receptor with collagenous structure expression) have been identified in some chronic liver inflammatory diseases (primary sclerosing cholangitis, primary biliary cholangitis, non-alcoholic steatohepatitis, and alcoholic hepatitis). IL-10 is a key immunosuppressive cytokine that limits proinflammatory responses by inhibiting antigen-presenting cell activation, reducing the production of crucial proinflammatory cytokines, suppressing antigen presentation, and promoting regulatory T-cell function and B-cell survival and antibody class switching. It also enhances tissue repair by limiting immune-mediated damage and can drive metabolic and transcriptional programs in macrophages toward anti-inflammatory phenotypes. MARCO, a scavenger receptor that binds pathogen- and damage-associated molecular patterns, helps to sequester proinflammatory ligands and suppress local immune activation. The development of MARCO-positive immunoregulatory macrophages is microbiota-dependent, indicating that commensal bacteria drive their induction to limit excessive inflammation at the gut–liver interface. The failure of this self-limiting circuit seems to promote hepatic inflammatory disease. A loss of hepatocytes and an increased ductular reaction are tightly associated with the accumulation of monocyte-derived macrophages and represent a prominent, shared immunological feature of inflammatory liver injury. Functional ablation of these macrophages can produce CVID-like inflammatory phenotypes in animal models, highlighting their importance in inflammation control. Although this periportal immunosuppressive macrophage subset has not been directly investigated in CVID-associated liver disease yet, its role in shaping the inflammatory microenvironment and in defense against gut-translocated microbes warrants systematic investigation [58,59]. These mechanisms are most likely intricate. Interestingly, some reports, and cases from our own experience, have described cryptogenic cirrhosis as a manifestation of liver disease in patients with CVID. Reports of cryptogenic cirrhosis in CVID are rare and only sparsely discussed in the literature [6,38,60,61,62]. However, further efforts are warranted to determine the prevalence of unexplained liver disease in this patient population and to elucidate the underlying pathophysiological mechanisms.

Altered coagulation and hypercoagulability are also relevant, as CVID patients with PSVD often present with thrombocytopenia and may have a prothrombotic state. Autoimmune thrombocytopenia is common in CVID and contributes to microvascular risk. The presence of thrombocytopenia and immune-mediated platelet destruction suggests that platelet dynamics are altered and may contribute to microvascular complications [63,64].

In PSVD, microvascular changes and the thrombotic risk arise from a combination of endothelial dysfunction, hypercoagulability, immune-mediated injury, and platelet activation, with gut-derived endotoxemia acting as a central driver of these processes [21,28,29,51,52].

In CVID, reduced naïve CD45RA+CD4+ T cells and the expansion of CD21^low^ B cells are markers associated with a higher risk of liver involvement, NRH and PH, indicating that specific immunophenotypes predispose to these vascular complications [45,64]. Their precise role in PSVD is not known. Genetic studies have identified mutations in immune-related genes in PSVD, and pathway analyses suggest that immune cell dysfunction—particularly involving T and B lymphocytes—may drive vascular remodeling and sinusoidal injury [46].

### 2.3. The Genetic Landscape of PSVD

Identifying the factors that predispose some of the CVID individuals to PSVD, while others remain unaffected is an unresolved question in the pathogenesis of CVID-associated hepatic complications. Despite the identification of at least 68 mutations contributing to CVID, a genetic etiology remains uncharacterized for the majority of patients. CVID is rarely monogenic, and, in most cases undefined, likely due to polygenic, oligogenic, or multifactoral inheritance patterns [13]. Furthernore, data elucidating the genetic interrelationship between CVID and its hepatic manifestations, including PSVD, is yet to be understood. Broad genomic investigations within these patient populations might provide critical insights regarding this topic.

A recent publication details the genetic variants implicated in PSVD [46]. Along with data published in the latest update of the IEI classification from the International Union of Immunological Societies Expert Committee, a common genetic landscape of IEI and PSVD can be identified (Table 2) [2,46]. In addition to data included in Table 2, a recent publication describes a de novo, dominant-negative (DN)-proteasome subunit beta type-8 (*PSMB8*) variant (p.G209R) that associates a clinical phenotype comprising of PSVD, infections, panniculitis, cytopenias and systemic inflammation [2,65,66].

Certain genetic mutations have been associated with isolated PSVD or with extrahepatic manifestations of patients with other systemic diseases and PSVD. Isolated PSVD was described in association with mutations of the different genes, including Potassium Calcium-Activated Channel Subfamily N Member 3 (*KCNN3*), Deoxyguanosine Kinase *(DGUOK)*, Familial Obliterative Portal Venopathy *(FOPV)*, GTPase IMAP Family Member 5 (*GIMAP5*), FCH And Double SH3 Domains 1 (*FCHSD1)*, TRNA Methyltransferase 5 (*TRMT5*), and Histidine Rich Glycoprotein (*HRG)* [46]. Interestingly, these gene mutations are predominantely expressed in immune cells, highlighting a critical knowledge gap, as the mechanistic link to hepatic vascular remodeling is still undefined. Most of these PSVD genes are not included in the IEI genetic testing panel. Expanding the testing of IEI patients to include genes associated with PSVD could improve patient risk stratification and possibly expand knowledge on this association of diseases.

### 2.4. Assessment of Liver Disease Associated with CVID

PSVD is a rare condition that causes PH and its severe complications, making it an essential prognostic factor impacting survival. The presence of liver disease in CVID, especially PSVD, indicates a poor prognosis. A recent large-scale study involving 587 patients with PSVD, which followed the natural course and survival, showed that, when these patients are followed in specialized hepatology centers, the prognosis is good, with a transplant-free survival rate of 83% at 5 years and 72% at 10 years. The diagnosis of PSVD is often established in late stages, when PH-related complications occur [19]. The mean onset of PSVD and of PH in CVID patients is unknown, underscoring that screening for liver disease should be broad.

#### 2.4.1. Laboratory Parameter Evaluation

Performing liver tests is a crucial first step in the initial screening and diagnosis of liver disease. However, RNH can progress longtime with normal liver test results, so these tests do not help screen for PSVD [21]. Some CVID patients with liver disease may present with thrombocytopenia and normal liver tests [23].

Cases of CVID and seronegative autoimmune hepatitis with normal serum IgG levels have been reported, which is contrary to the typical pattern of elevated IgG, explained by the characteristic hypogammaglobulinemia of this immunodeficiency [36]. Therefore, a laboratory diagnosis of AIH in these patients is difficult. In these cases, liver biopsy is particularly important.

Liver tests, especially alkaline phosphatase, can be elevated in some CVID patients. The presence of hepatocellular syndrome warrants further investigations with hepatic autoantibody markers. If these tests are severely altered, a liver biopsy is recommended to exclude seronegative AIH. It is advised to evaluate these parameters at least annually if the initial assessment is within normal limits. Since laboratory evaluation can be normal in PSVD, imaging investigations are recommended to complete the assessment of CVID patients [19,20,21,22].

#### 2.4.2. Imaging Assessment

Abdominal ultrasonography, hepatic and splenic elastography, and abdominal MRI can reveal structural, anatomic, and hemodynamic alterations of the spleen–liver axis. Because PH is a serious complication, ultrasonography and elastography are first-line investigations [19]. Splenomegaly is the most frequently encountered feature in patients with CVID complicated by NRH and NCPH [19,23,36], but this is a non-specific finding. In CVID patients, splenomegaly is often present as a feature of the benign lymphoproliferation characteristic of this immunodeficiency, together with hepatomegaly or lymphadenopathy. ESID reports splenomegaly in 25% of patients, while French CVID cohort studies show a splenomegaly prevalence of 38% [22,67]. A spleen diameter of at least 16 cm is predictive of PH [19,22,23]. PH in CVID is easily overlooked in sonography due to its non-cirrhotic nature. Elastography demonstrates increased liver stiffness. There is no consensus on the liver stiffness threshold predictive of PH in non-cirrhotic patients. Some studies report a cut-off value of 11.2 kPa, while others show that most patients with liver stiffness > 6.5 kPa—and all patients with values > 20 kPa—have PH [19,20,21,22]. Measurement of splenic stiffness may provide additional valuable data. Several studies demonstrated increased splenic stiffness in patients with PSVD without CVID [67,68,69,70,71]. Future studies are needed to validate this information. CT scanning can identify signs of PH, such as caliber changes of venous collaterals and intrahepatic vessels, especially in peripheral branches of the portal vein, the latter being a sign of obliterative venopathy [72]. Ultrasound is recommended every 6 months and elastography every 1–2 years for the early detection of PH [73]. Another valuable recommended test is liver biopsy [19,27].

#### 2.4.3. The Hepatic Venous Pressure Gradient

The Hepatic Venous Pressure Gradient (HVPG) is a crucial hemodynamic measurement used to quantify the degree of PH. HVPG in patients with PSVD is typically normal or only mildly elevated, often measuring less than 10 mmHg, even in the presence of clinically significant PH. As PSVD is characterized by presinusoidal or sinusoidal PH, HVPG does not reliably reflect true portal pressure in these patients. The American Association for the Study of Liver Diseases (AASLD) specifically notes that HVPG measurement is inaccurate for estimating portal pressure in PSVD, and clinical and endoscopic evaluation for PH complications is recommended instead. Liver biopsy and imaging remain essential for diagnosis and assessment in PSVD [74,75].

#### 2.4.4. Liver Biopsy

Liver biopsy is the gold standard for diagnosing NRH. In 2019, Vascular Liver Disease Group (VALDIG) proposed a histology diagnostic criterion for PSVD: “CVID and NRH, and absence of histologic cirrhosis”. Thus, liver biopsy is the cornerstone of diagnosis and management [27].

The procedure can be performed percutaneously, but transjugular or laparoscopic approaches may be used in patients with coagulopathy or significant trombocytopenia, which are frequent in CVID with PH [76].

In CVID, consensus among expert centers is that liver biopsy is critical for confirming the diagnosis and characterizing the pattern of liver injury [21,37]. Biopsy findings are essential for confirming the diagnosis and distinguishing CVID-related liver disease from other etiologies [21,77]. The identification of NRH, pericellular fibrosis, and the absence of plasma cells supports a diagnosis of CVID-related liver disease and excludes other etiologies. Histopathological findings from liver biopsy in CVID with liver involvement directly influence management by guiding diagnosis, risk stratification, and therapeutic decisions, and they have major prognostic implications due to their association with PH, fibrosis progression, and increased mortality.

### 2.5. Treatment of CVID and PSVD

Given the lack of standardized guidelines and prospective data, the early detection and close monitoring of liver disease in CVID are critical. A consensus on the optimal management of CVID-associated liver disease is lacking [22,37]. Table 3 summarizes the various therapeutic options available for managing CVID-related liver involvement.

The rate of mortality in CVID patients with liver disease is significantly higher than in those without hepatic involvement. Large cohort studies report in-hospital mortality rates of 11% for CVID admissions with hepatic complications compared to 4% without. All-cause mortality in CVID-related liver disease is 26.4% versus 11% in those without liver involvement, with PH and increasing fibrosis being key predictors of death. The presence of NRH and progressive fibrosis, along with PH, is linked to increased morbidity and mortality in CVID [22,37,89]. Fibrosis can progress even in young patients, and the coexistence of NRH and fibrosis often reflects a more aggressive disease course [21,37,39]. Semiquantitative scoring systems and image analysis techniques are increasingly used to assess the extent of fibrosis, elastosis, and immune cell infiltration, and these findings may be correlated with clinical parameters such as liver stiffness and PH [39]. Other factors associated with poor prognosis and higher risk include high-grade varices, presence of ascites, serum bilirubin, albumin, and creatinine levels, age, and comorbid chronic diseases [19,21].

Most deaths in this population are attributable to infections and sepsis. This increased susceptibility is due to the combination of impaired humoral immunity and the additional immune dysregulation associated with hepatic involvement, particularly PH and NRH [22,28,90]. These patients frequently exhibit lymphopenia, thrombocytopenia, and reduced naïve CD4+ T cells, which further compromise host defense mechanisms and predispose to severe, recurrent infections [22,23,45,91]. The high morbidity and mortality underscore the importance of multidisciplinary care and prompt intervention for infectious complications in this high-risk group.

Immunoglobulin replacement therapy is the cornerstone intervention shown to reduce infection rates and improve survival in patients with CVID, including those with liver disease. Both intravenous and subcutaneous immunoglobulin can be used, with dosing individualized to maintain infection-free status. Early initiation and adequate dosing are critical for reducing major infections and mortality [6,33,78,79].

Antibiotic prophylaxis may be considered in select CVID patients with advanced liver disease who meet criteria for cirrhosis-related infection risk, such as those with PH or prior spontaneous bacterial peritonitis. In these cases, regimens such as daily oral norfloxacin, ciprofloxacin, or trimethoprim–sulfamethoxazole are used until liver transplantation or death, as recommended for cirrhotic patients by the AASLD and supported by the medical literature [80]. However, routine prophylaxis in CVID without these specific risk factors is not established. Antimicrobial prophylaxis in CVID patients with liver disease is not universally indicated and should be tailored to individual risk, with close monitoring for adverse effects and resistance. Antifungal prophylaxis is not routinely indicated unless there is additional risk (e.g., profound neutropenia or ICU admission), and should be guided by local epidemiology and individual risk factors.

Treatment of CVID-related liver disease has two main components: disease-directed therapy addressing underlying drivers to prevent progression, and supportive treatment for NCPH [19].

Immunosuppressive agents may be used for autoimmune/inflammatory liver involvement, but evidence is heterogeneous and individualized [22,74].

Cause-directed treatment is guided by histology. In cases with granulomatous disease, immunosuppressants (steroids, anti-Tumor Necrosis Factor (TNF) biologics) have been reported to be effective [67]. Immunosuppressants (corticosteroids, azathioprine, 6-mercaptopurine) also provide clinical benefit in NRH and rapidly progressive liver disease. Rituximab, mycophenolate mofetil, azathioprine, and corticosteroids can be life-saving when NRH is associated with severe lymphoproliferative features such as GLILD [37,72,74,75]. A case of NRH with enteropathy improved on oral budesonide [61]. Rare cases of NRH and PH have been associated with thiopurine therapy, particularly in inflammatory bowel disease (IBD) patients without CVID. Thioguanine seems to be associated with a higher risk than other thiopurines, suggesting that the risk of NRH may be dose-dependent [20,92,93,94,95].

The treatment of NCPH should begin at the first signs of disease. NCPH development appears to reflect the slow progression of liver injury in these patients; onset has been reported a median of 11.8 years after CVID diagnosis [19]. Variceal hemorrhage is a feared complication. Primary prophylaxis includes nonselective beta-blockers, preferably carvedilol, or endoscopic variceal ligation. In cirrhosis-related PH, carvedilol has demonstrated superior reduction in HVPG compared with propranolol [19,81,82,83]. There is no consensus on the single most effective primary prophylaxis method [20,23]. Some studies favor primary prophylaxis by endoscopic ligation, while others report similar clinical benefit with nonselective beta-blockers [83,96,97]. Comparison is difficult; a large cohort of CVID patients with PSVD—most treated with nonselective beta-blockers—showed a relatively low bleeding risk of 15% at 5 years, supporting the efficacy of beta-blocker therapy. The risk of variceal bleeding in CVID with liver disease is similar to that in cirrhosis; however, short-term mortality is low (3.4%) in CVID, likely reflecting better hepatic reserve than in cirrhosis without CVID. Secondary prophylaxis with nonselective beta-blockers plus endoscopic variceal ligation is highly effective, with a recurrence of gastrointestinal bleeding at 5 years documented in <20%—a more favorable outcome than in cirrhosis [19]. Anticoagulation has also been proposed, supported by clinical benefit observed in these patients, similar to that seen in cirrhotic patients, suggesting that coagulation abnormalities contribute to PSVD pathogenesis.

Failure to control variceal bleeding and refractory ascites may require the placement of a transjugular intrahepatic portosystemic shunt (TIPSS) [19,20,21,86], which effectively reduces PH. While this method has proven effective in reducing PH and splenomegaly, it is associated with a high risk of sepsis. Sepsis-related mortality or multiple organ failure secondary to TIPSS has been reported up to 4 years post-placement [6,23,86,98]. The presence of persistent enteropathy is hypothesized to be a contributing risk factor. Furthermore, a consensus on antibiotic prophylaxis in these cases is currently lacking. Experts recommend antimicrobial prophylaxis (e.g., norfloxacin 400 mg daily) for all CVID patients with TIPS, given the heightened risk of Gram-negative sepsis associated with bacterial translocation [80]. A critical consideration involves the potential for increased systemic adverse events of medications (e.g., budesonide) due to TIPS-induced hepatic bypass.

Liver transplantation is life-saving for patients with progressive disease despite conventional therapy. A recent large study reported survival of 97% at 1 year and 83% at 5 years among 50 transplanted CVID–PSVD patients. Conversely, lack of transplant in high-risk CVID with NCPH is associated with reduced survival, down to ~60% at 5 years [19]. These data support the benefit of liver transplantation in CVID with severe PH.

## 3. Future Directions

Despite the increased knowledge of liver disease in CVID, several critical questions remain unresolved. The precise immunopathogenic mechanisms remain incompletely elucidated. The predominant expression of PSVD-associated gene mutations in immune cells highlights a critical knowledge gap, as the mechanistic link to hepatic vascular remodeling is still undefined. The interplay between immune-mediated injury, genetic susceptibility, and environmental or infectious triggers requires further clarification.

Moreover, the unmet needs in the diagnosis, management, and treatment of CVID related liver diseases, including PSVD, are substantial.

In diagnosis, there is a lack of sensitive and specific biomarkers for the early detection of liver involvement in CVID. Standard liver biochemistry and ultrasound often fail to identify early or subclinical disease, while transient elastography and splenomegaly may be more sensitive but are not universally adopted. Liver biopsy remains essential for definitive diagnosis. However, there is no consensus on timing and indications, and non-invasive alternatives are yet to be validated.

In management, there are no evidence-based, disease-specific guidelines for CVID-related liver disease, including PSVD. The heterogenity of liver pathology (NRH, lymphocytic infiltration, fibrosis) complicates standardized management, and risk stratification tools are lacking.

In treatment, IgRT is universally recommended, but there is no established therapy for liver-specific complications. Immunosuppressive and immunomodulatory agents are used off-label with variable efficacy and significant infection risk, and randomized control trials are absent. There is also a need for validated protocols for infection prohylaxis and anticoagulation in patients with PH and thrombosis.

## 4. Conclusions

Liver disease seems to be a common finding in CVID cohorts, with PSVD being the most frequent manifestation. Proactive screening for CVID-associated liver disease is warranted, as complications of portal hypertension, which are potentially preventable, frequently serve as the inaugural clinical signs. Although the understanding of hepatic involvement in CVID has advanced substantially, further research is required to address remaining knowledge gaps and to establish evidence-based guidelines for diagnosis, monitoring, and management. International, multicenter collaboration is essential to generate the robust data needed to inform these recommendations.

## Figures and Tables

**Table 2:** Genetic overlap between IEI and PSVD.

Major Category						
I. Bone marrow failure with immune deficiency						
IEI	Genetic defect	Inheritance	Gene OMIM	T cells, B cells, other affected cells	Associated features	Variant associated with PSVD [50]
DKCA2	*TERT*	AD/AR	187,270	Normal to low T cells, B cells HSC	Bone marrow failure, pulmonary and hepatic fibrosis, nail dystrophy, leukoplakia, reticulate skin pigmentation; microcephaly, neurodevelopmental delay	K570-S368F-Gly135Glu-Val170Met-His983Tyr-Lys1050Asn
DKCX1	*DKC1*	XL	305,000		Bone marrow failure, pulmonary and hepatic fibrosis, nail dystrophy, leukoplakia, reticulate skin pigmentation; microcephaly, neurodevelopmental delay	IVS1 + 592 C>G
DKCB5	*RTEL1*	AR	615,190	Low	Nail dystrophy, leukoplakia, bone marrow failure, severe B-cell immunodeficiency, intrauterine growth retardation, growth retardation, microcephaly, cerebellar hypoplasia, and esophageal dysfunction	Arg1010
Coats plus syndrome	*CTC1*	AR	617,053	Not reported	Intrauterine growth retardation, premature aging, pancytopenia, hypocellular bone marrow, gastrointestinal hemorrhage due to vascular ectasia, intracranial calcification, abnormal telomeres	c.833G>T–c.841T>C
	*STN1*	AR	613,129	Normal		c.404G>C–C.469G>T
II. Autoinflammatory disorders Type 1 Interferonopathies						
TREX1 deficiency, Aicardi-Goutieres syndrome 1 (AGS1)	*TREX1*	AR/AD	606,609	Not assessed Functional defect: Intracellular accumulation of abnormal ss DNA species leading to increased type I IFN production	Classical AGS, SLE, FCL	V235Gfs*6
III. Diseases of Immune Dysregulation, Autoimmunity with or without Lymphoproliferation						
Prolidase deficiency	*PEPD*	AR	613,230	Normal circulating T and B cells, peptidase D functional defect	Autoantibodies common, chronic skin ulcers, eczema, infections	c.671+2 T > G (likely pathogenic) c.1354G>A; p.Glu452Lys and c.671+3_671+11del (VUS)

AD: autosomal-dominant; AGS: Aicardi–Goutieres syndrome; AGS1: Aicardi-Goutieres syndrome 1; AR: autosomal recessive; *CTC1*: Conserved Telomere Maintenance Component 1; DKCA2: Dyskeratosis Congenita, Autosomal Dominant 2; *DKC1*: Dyskerin Pseudouridine Synthase 1; DKCX1: dyskerin pseudouridine synthase 1; DKCB5: Dyskeratosis Congenita, Autosomal-Recessive 5; DNA: deoxyribonucleic acid; FCL: familial chilblain lupus; HSC: hematopoietic stem cells; IEI: inborn error of immunity; IFN: interferon; OMIM: Online Mendelian Inheritance in Man; *PEPD*: Peptidase D; PSVD: Porto-sinusoidal vascular disease; *RTEL1*: Regulator of Telomere Elongation Helicase 1; SLE: systemic lupus erythematosus; *STN1*: member of the CTC1-STN1-TEN1 or CST complex; *TERT*: telomerase reverse transcriptase; TREX1: Three-Prime Repair Exonuclease 1; VUS: variants of unknown significance; XL: X-linked.

**Table 3:** Therapeutic options available for managing CVID-related liver involvement.

Intervention	Rationale
IgRT [6,33,78,79]	- infection-free status, prevention of end-organ damage, and improved survival - indicated in all CVID patients with significant antibody deficiency
Antimicrobial prophylaxis [80]	- indicated in CVID patients with advanced liver disease (e.g., PH, advanced fibrosis, prior spontaneous bacterial peritonitis, after TIPSS), in those with recurrent or severe infections despite adequate IgRT, or those receiving additional immunosuppression for autoimmune/inflammatory liver complications, and high additional risk of infections (e.g., profound neutropenia or ICU admission) - the benefit of routine prophylaxis must be balanced against the risk of multidrug-resistant organisms and drug-related toxicity
Immunosuppressive and immunomodulatory therapy (e.g., corticosteroids, azathioprine, mycophenolate mofetil, biologics) [22,74]	- can be considered for autoimmune, granulomatous or inflammatory liver involvement, as well as in cases with severe lymphoproliferative features (GLILD); evidence is limited - treatment is individualized - used cautiously due to infection risk
Nonselective beta-blockers [19,81,82,83]	- primary prophylaxis of NCPH * and variceal bleeding (preferably carvedilol, due to superior reduction of HVPG)
Endoscopic variceal ligation [19]	- primary prophylaxis of NCPH * and variceal bleeding - indicated for high-risk varices
Anticoagulants [84,85]	- play a central role in the management of thrombotic complications (particularly in portal vein thrombosis, and in other thrombotic events) - patient selection shoul be individualized; bleeding risk, while a concern, is not substantially increased when patients are appropriately selected and gastroesophageal varices are managed prior to therapy - their role in PSVD management is unclear
Transjugular intrahepatic portosystemic [19,20,21,86]	- reduces PH - indicated in refractory ascites and in failyre to control variceal bleeding (consider antimicrobial prophylaxis)
Liver transplantation [19,21,37,87,88]	- life-saving for patients with progressive disease despite conventional therapy
Other supportive care measures	- regular monitoring for malignancy - multidisciplinary management of organ-specific complications

* there is no consensus on the single most effective primary prophylaxis method. CVID: common variable immunodeficiency; GLILD: granulomatous and interstitial lymphocytic pulmonary disease; HVPG: hepatic venous pressure gradien; IgRT: immunoglobulin replacement therapy; NCPH: non-cirrhotic portal hypertension, PH: portal hypertension; PSVD: Porto-sinusoidal vascular disease; TIPSS: transjugular intrahepatic portosystemic shunt.

## Data Availability

No new data were created or analyzed in this study. Data sharing is not applicable to this article.
